# A Vision-Based Dynamic Rotational Angle Measurement System for Large Civil Structures

**DOI:** 10.3390/s120607326

**Published:** 2012-05-30

**Authors:** Jong-Jae Lee, Hoai-Nam Ho, Jong-Han Lee

**Affiliations:** 1 Department of Civil and Environmental Engineering, Sejong University, Seoul 143-747, Korea; E-Mail: namhohoai@sju.ac.kr; 2 Research & Engineering Division, POSCO E & C, Incheon 406-732, Korea; E-Mail: jonghan.lee@poscoenc.com

**Keywords:** rotation angle, vision-based system, displacement measurement, flexible structures

## Abstract

In this paper, we propose a vision-based rotational angle measurement system for large-scale civil structures. Despite the fact that during the last decade several rotation angle measurement systems were introduced, they however often required complex and expensive equipment. Therefore, alternative effective solutions with high resolution are in great demand. The proposed system consists of commercial PCs, commercial camcorders, low-cost frame grabbers, and a wireless LAN router. The calculation of rotation angle is obtained by using image processing techniques with pre-measured calibration parameters. Several laboratory tests were conducted to verify the performance of the proposed system. Compared with the commercial rotation angle measurement, the results of the system showed very good agreement with an error of less than 1.0% in all test cases. Furthermore, several tests were conducted on the five-story modal testing tower with a hybrid mass damper to experimentally verify the feasibility of the proposed system.

## 1. Introduction

In civil engineering, damage in a structure generally causes a local increase in flexibility, which depends on the extent of the damage. However, it is very difficult to evaluate the structural condition and performance under today's traffic and to decide which structural components need to be retrofitted or replaced by new structural members to optimize the available budget. Angular orientation is one of the key parameters in structural health monitoring [1] and an early warning of potential damages. Generally, rotation angle measurement has been successfully applied in many applications such as displacement measurement of high-rise buildings [2], examining deformations of bridge spans [3], ground movement monitoring [4,5], automobiles [6], *etc.* Recently, there were some studies focusing on development of rotation measurement systems using a gyroscope [7,8].

Rotation angle measurement systems are widely used nowadays to monitor deformations of large-scale civil structures such as long-span bridges, tunnels, dams, high-rise buildings, *etc.* In 2010, Park *et al.* [2] proposed a displacement measurement system for civil structures using a partitioning approach. Calculation of rotation angle is only an intermediate step to obtain the total displacement of the measurement point at a far distance. Only the basic scheme was verified through static laboratory tests, so the efficiency and feasibility for dynamic measurements are still under scrutiny. More recently, Jeon *et al.* [3] introduced in 2011 a paired structured light for displacement measurement of structures. This system could simultaneously obtain the displacement and rotation of the measurement point. However, the system had a complicated setup and required a transformation matrix to obtain the final results.

Some current commercial contact-type tilt-meter sensors can provide a resolution up to 0.002 degree, as shown in Table 1. Basically, a commercial rotation angle measurement system usually consists of a tilt sensor, data cable, data logger, and data processing software, so the total cost of the system is still expensive. The total cost of a commercial rotation angle measurement system usually varies from several hundred to several thousand US dollars, depending on a specific application.

Painter and Shkel [1] have briefly reviewed some typical contact-type direct angle measurement technologies and pointed out their disadvantages. Inclinometers and magnetometers are highly sensitive to linear acceleration and environmentally induced magnetic fields, respectively. In addition, inclinometers only measure rotations about axes perpendicular to gravity, while magnetometers can only capture angular changes about axes parallel to gravity. Gyroscopes can produce large errors in as short as an hour, due to integration bias and noise.

The objective of this study is to develop a single-point rotational angle measurement system for large-scale civil structures using a multi-point vision-based system with high resolutions. Compared to commercial rotation angle measurement systems, the proposed system can measure static and dynamic rotational angles with high resolutions. Moreover, the system can be easily expanded to multi-point rotation angle measurement using the partitioning approach [2]. To verify the performance of this system, several laboratory and field tests were carried out on a three-story steel frame model, and on a five-story modal testing tower with a hybrid mass damper (HMD).

## 2. A Vision-Based Rotation Angle Measurement System

**Figure 1.**
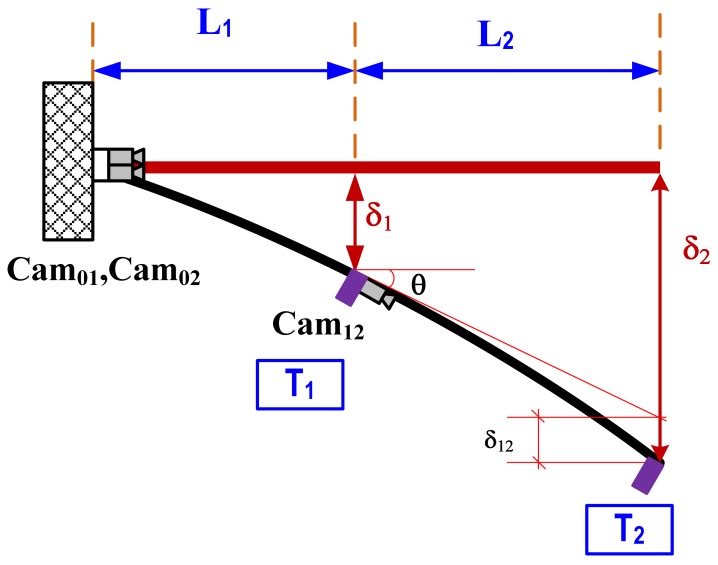
Vision-based dynamic rotation angle measurement.

The configuration of the proposed system is provided in Figure 1. The initial assumption is that two camcorders, Cam_01_ and Cam_02_, are placed at the fixed location base of the structure or beyond the structure where there is no displacement and rotation (δ_0_ = 0, and θ_0_ = 0). Cam_01_ and Cam_02_ will track targets T_1_ and T_2_, respectively. Another camcorder (Cam_12_) will aim at target T_2_. Displacements δ_1_ and δ_2_ are directly measured by Cam_01_ and Cam_02_, and the displacement δ_12_ is the relative displacement of target T_2_ with respect to the tangential line of Cam_12_. Finally, rotational angle θ can be calculated as:

(1) $$\theta =\text{arctan}\;\left\{\frac{\delta_{2}-\delta_{1}-\delta_{12}}{L_{2}}\right\}$$

The multi-point vision-based system for dynamic real-time displacement measurement was introduced by Lee *et al.* [16]. To measure the structural displacement, a target panel needs to be attached on a desired location of a structure, and then camcorders capture the images of the target panel from a remote distance. Next, the images were streamed into the PC, and the software calculated the displacement of the target by applying image-processing techniques with pre-measured calibration parameters.

**Figure 2.**
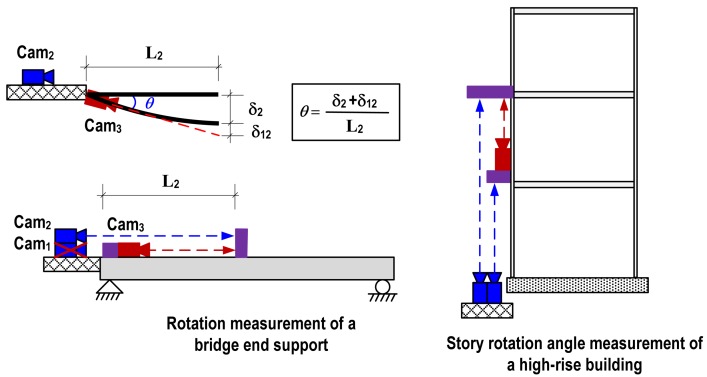
Typical application examples.

The proposed vision-based rotational angle measurement system has two key assumptions: fixed starting point and single curvature within the measurement range. For large-scale civil structures such as bridges and high-rise buildings, these assumptions can be easily satisfied. The starting point can be located beyond the structure or at the ground level as shown in Figure 2. Since the deformation of large structures is mainly depending on the several lower modes, the assumption on single curvature within a reasonable range can be easily satisfied. Figure 2 illustrates example measuring schemes of rotation angle at a bridge end support and story rotation of a high-rise building. In rotation measurement of the bridge end support, the 1st camera is omitted due to no displacement at the end support. The 2nd camera installed at the fixed location where is no displacement and rotation tracks the 2nd target, and then the 3rd camera placed at the end support also aims at the 2nd target.

## 3. Laboratory Verifications

**Figure 3.**
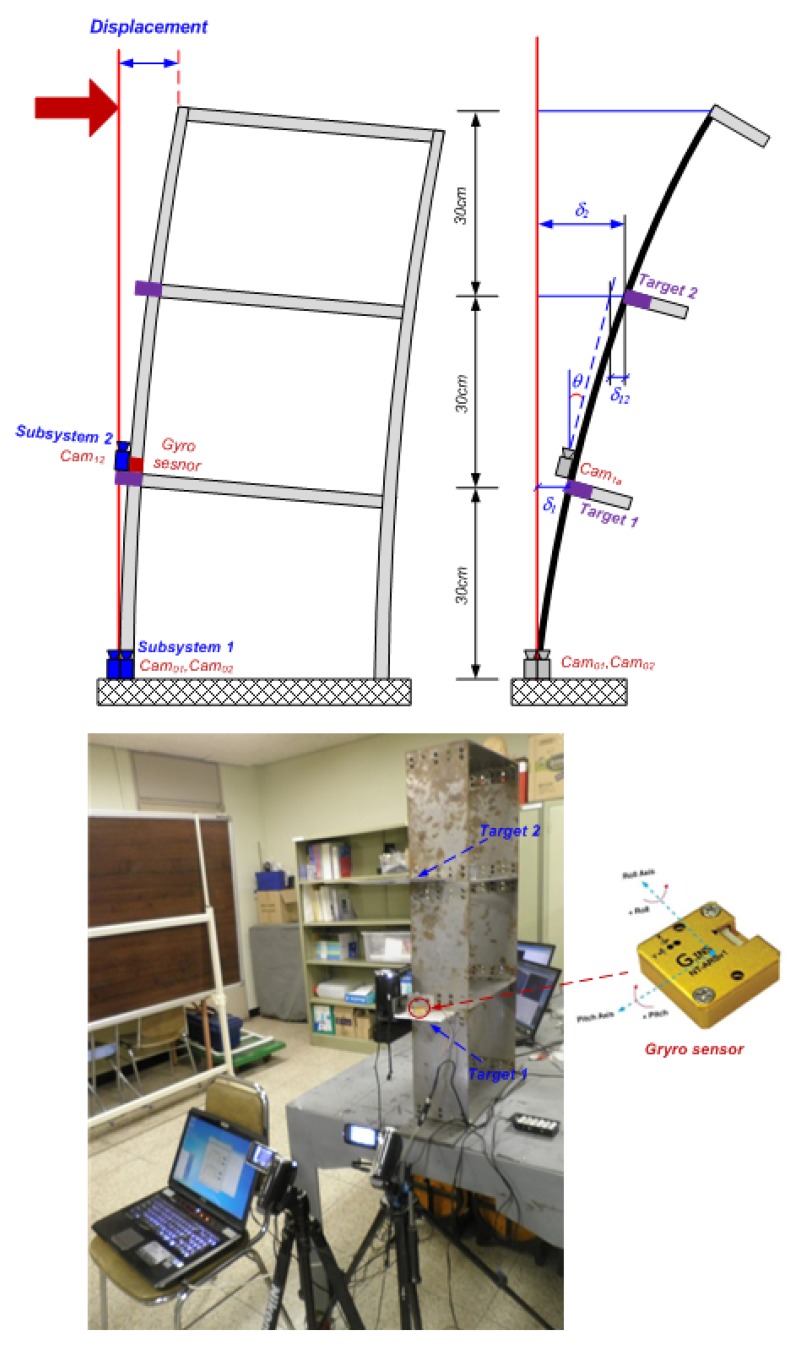
Experimental setup.

Two types of laboratory tests were conducted on a three-story steel frame model. The first tests focused on the accuracy of the proposed system in a static case, and the second assessed the feasibility of the system in a dynamic case. The detailed test settings are provided in Figure 3. We deployed commercial camcorders with 40 times optical zooming capability, a resolution of 640 × 480 pixels, and the frame rate of 30 frames per second (fps). Optical equipment (such as lenses, and cameras, *etc.*) and target size play important roles in a target-based measurement system using image processing. The most important issue to select the optical devices is that they should have low image distortion. For target size selection, it is necessary to roughly estimate the maximum displacement at the measurement point and performance of optical equipment to obtain a reasonable size of the target. Thus the target size used in this test is 10 mm × 10 mm. To suggest reference data, a Gyro sensor NT-ARSv1 [17], was installed at the same location of Target 1, allowing direct observation of rotational angle. The sensor has a resolution of 0.001 radian (or 0.057 degree) and sampling rate of 100 Hz.

**Figure 4.**
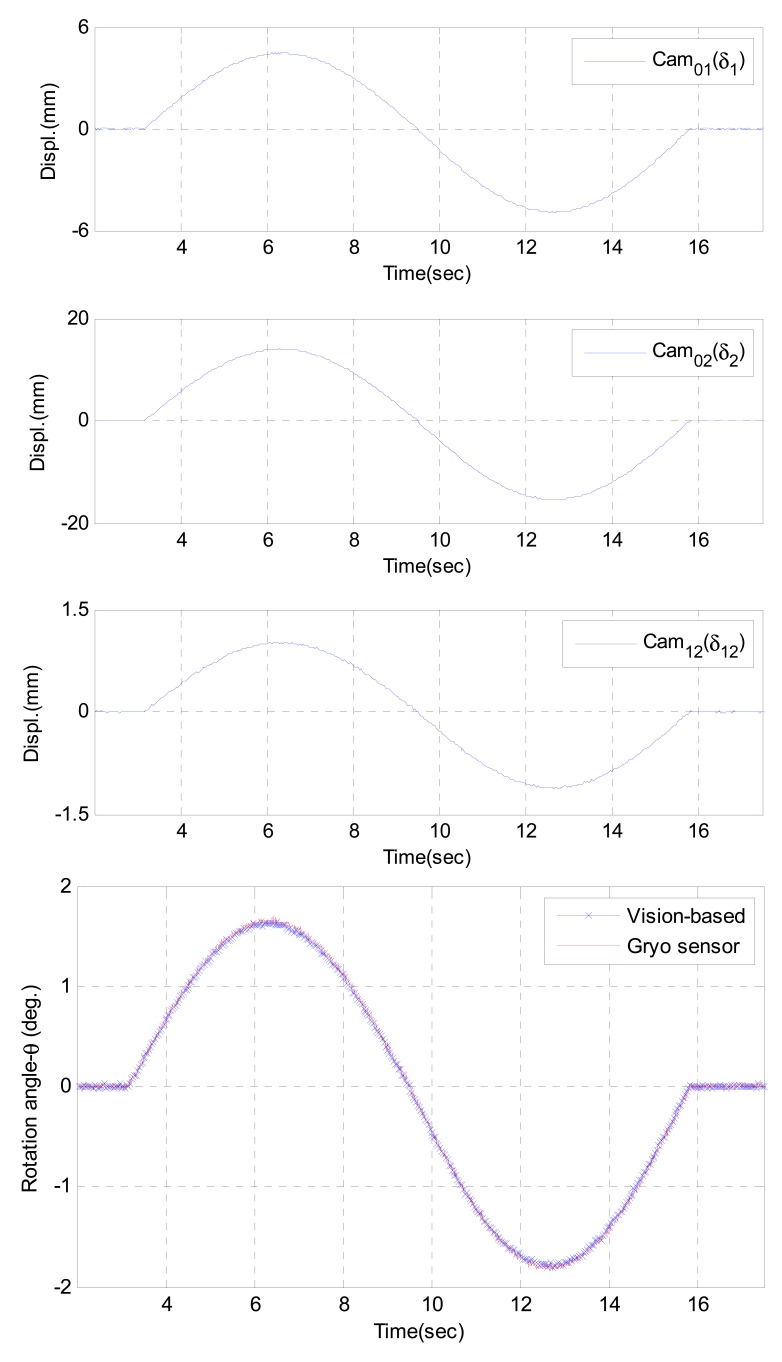
Dynamic test results.

The tests were conducted by applying a series of artificial displacements at the top of the structure. Three cameras captured the deflections at the Target 1, and Target 2; then all the information were wirelessly sent to Master PC to calculate the rotational angle (θ) at Target 1 using Equation (1). The results of static and dynamic tests are given in Table 2 and Figure 4, respectively. The average of errors (differences) in the static case is 0.68%, the outputs of the proposed system showed very good agreement with the commercial sensor outputs. Similarity, the average error in dynamic tests is less than 1.0% in root-mean-square (RMS) level, and the results were also very close with Gyro sensor outputs.

## 4. Testing on a Five-Story Modal Tower

### 4.1. Testing Setup

**Figure 5.**
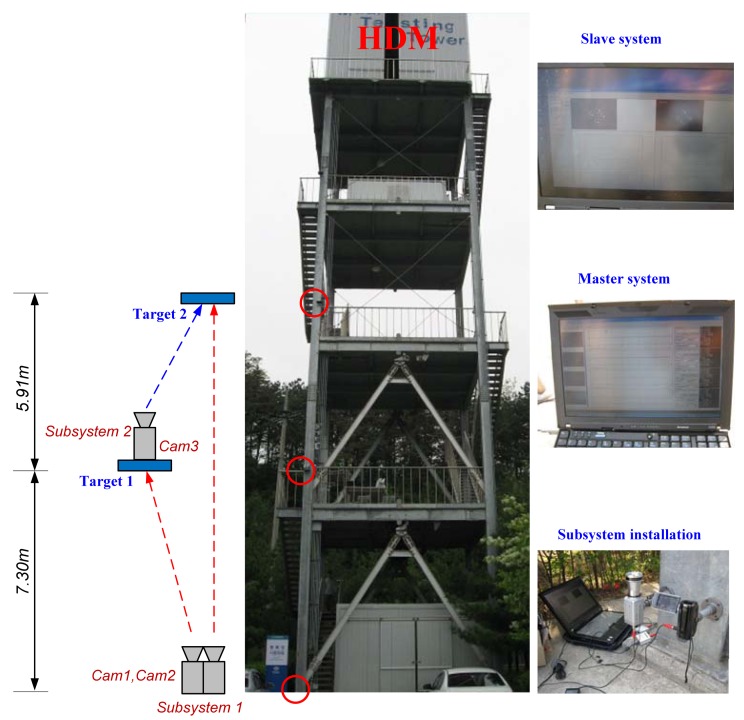
Field-test setup.

The proposed system was verified through a full-scale implementation on a five-story modal testing tower with an HMD on the top floor. Figure 5 shows the test structure and experimental setup. The cameras were divided into two groups: Subsystem 1 and Subsystem 2. Subsystem 1 was located at the ground floor, and Subsystem 2 was installed at the second floor. All the measured data of Subsystems 1 and 2 were wirelessly transmitted to the master PC. To excite the test structure, we used an HMD by applying two excitation methods: sinusoidal and random excitation.

### 4.2. Results and Discussion

**Figure 6.**
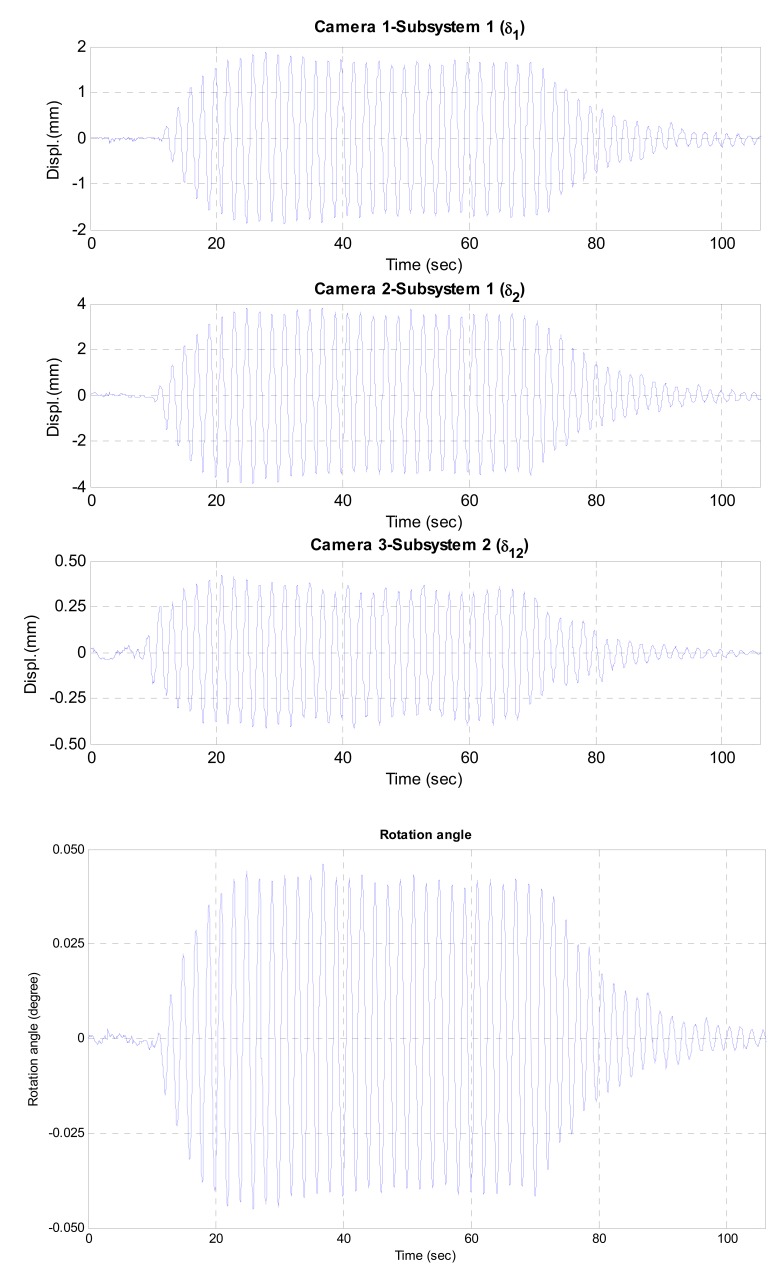
Measured and estimated data under sinusoidal excitation.

**Figure 7.**
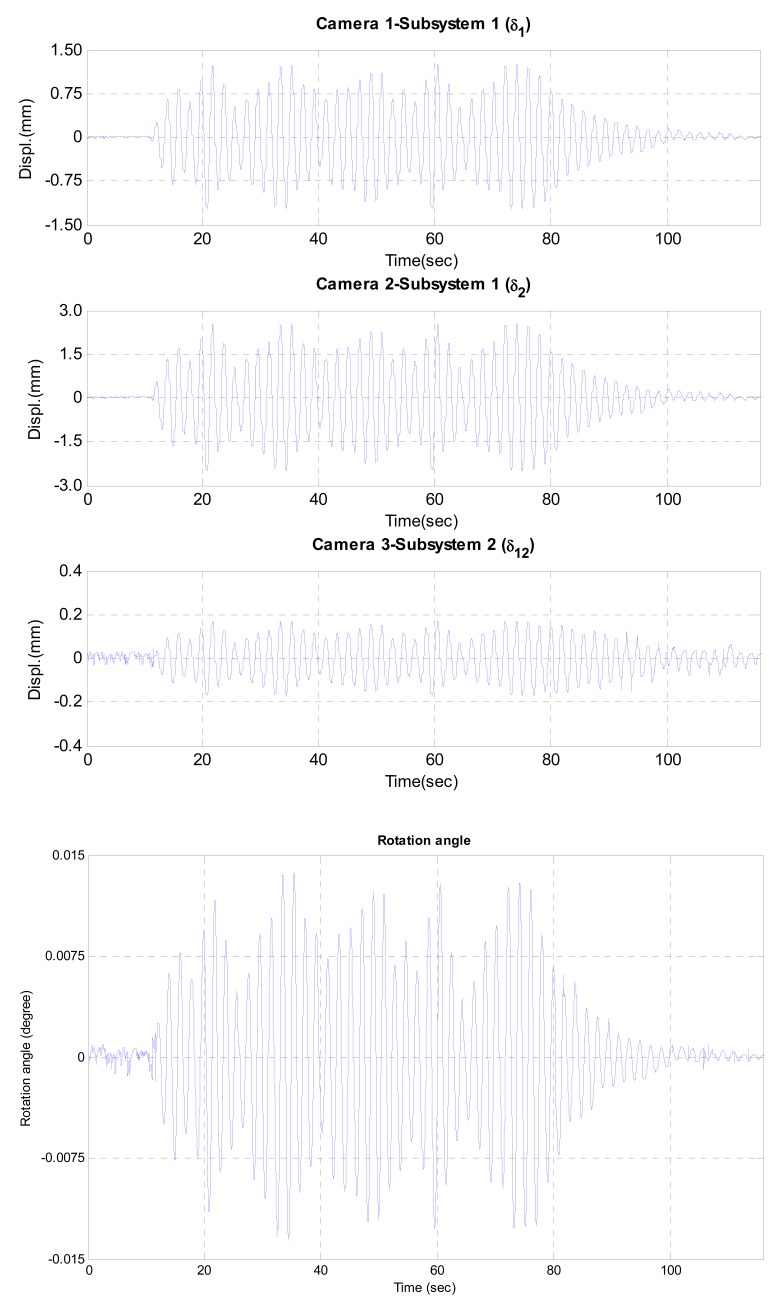
Measured and estimated data under random excitation.

Figures 6 and 7 show the test results including the displacement data measured by three cameras and the estimated dynamic rotational angle. In two test cases, the maximum rotational angles were less than 0.045° and 0.015° under sinusoidal and random excitations, respectively. The estimated rotational angle exhibited very good dynamic resolution of 10^−4^ degree. From those results, it can be concluded that the proposed vision-based system can measure the dynamic rotational angle of a flexible structure very accurately.

## 5. Conclusions

In this study, we have developed a single-point vision-based system for rotation angle measurement which is applicable to large-scale civil structures using commercial PCs, commercial camcorders, low-cost frame grabbers, and a wireless LAN router. The system can easily overcome the angle restrictions of inclinometers and magnetometers, and the large errors of gyroscopes in the short period of time due to integration bias and noise. In addition, measurement points can be expanded without difficulty using the partitioning approach [2]. The feasibility of the proposed system has been verified through laboratory and field tests. The results of laboratory tests showed very good accuracy compared to a commercial sensor with a maximum error of 1.0%. From the full-scale implementation of the five-story modal tower, it is observed that the proposed system can measure the dynamic rotational angle with a high resolution of 10^−4^ degree. In conclusion, the proposed vision-based rotational angle measurement system provides high accuracy and an effective alternative to measure static and dynamic rotational angle of large civil structures with high resolution.

## Figures and Tables

**Table 1.** Commercial tilt sensors.

**Device name**	**Measuring range (deg.)**	**Resolution (deg.)**	**Sampling rate (Hz)**	**Country**

IK360 [9]	±180	0.010	100.0	UK
M3 [10]	±70	0.010	3.3	Korea
SX 41100 [11])	±70	0.014	10.0	France
Portable Tiltmeter SC [12])	±53	0.002	×	USA
MNS-45-D2 [13]	±45	0.100	10.0	Germany
ARN-INC2D [14]	±40	0.010	*	India
ZCT215M-LBS-BUS-B1-30 [15]	±15	0.010	2.0	China

× unavailable data;

* static mode.

**Table 2.** Static test results.

**Test No.**	**δ_1_ (mm)**	**δ_2_ (mm)**	**δ_12_ (mm)**	**θ (deg)**	**Gyro sensor (deg)**	**Difference (%)**
1	4.45	13.88	1.05	1.601	1.592	0.57
2	5.65	16.85	1.51	1.851	1.865	0.75
3	6.41	18.99	2.19	1.984	1.973	0.56
4	6.81	20.19	2.31	2.114	2.128	0.66
5	8.16	24.42	2.42	2.640	2.663	0.86
